# Effective Transcriptional Induction of the CARMN/miR-143/145 Complex Locus in Smooth Muscle Cells Using CRISPR Activation

**DOI:** 10.1161/ATVBAHA.124.322353

**Published:** 2025-07-31

**Authors:** Francesca Vacante, Sandra Sanchez-Esteban, Themistoklis M. Tsarouchas, Julie Rodor, Matthew Bennett, Melissa S. Carroll, Abdelaziz Beqqali, Andrew H. Baker

**Affiliations:** Queens Medical Research Institute, British Heart Foundation Centre for Cardiovascular Sciences, University of Edinburgh, Scotland (F.V., S.S.-E., J.R., M.B., A.B., A.H.B.).; Centre for Regenerative Medicine, Institute for Regeneration and Repair, The University of Edinburgh, Edinburgh BioQuarter, Scotland (T.M.T.).; Now with Stanford University School of Medicine, CA (F.V., T.M.T.).; School of Cardiovascular and Metabolic Medicine and Sciences and British Heart Foundation Centre of Research Excellence, King’s College London, United Kingdom (M.S.C.).; Department of Pathology, Cardiovascular Research Institute, Maastricht School for Cardiovascular Diseases, Maastricht University, The Netherlands (A.H.B.).

**Keywords:** cardiovascular diseases, Cis-activation, microRNAs, RNA, long noncoding, smooth muscle cell

Over the past decade, advancements in understanding noncoding RNAs (ncRNAs) have revealed their critical role in regulating gene networks in cardiovascular development and disease. MicroRNAs (miRNAs) and long ncRNAs (lncRNAs) collectively regulate gene expression and cellular function. Their loci are often complex, with many microRNAs embedded within long ncRNA host genes. Examples include miR503HG/miR-503/miR-424, H19/miR-675, and CARMN/miR-143/miR-145. This presents significant challenges in understanding their regulation and in developing therapeutic strategies to precisely modulate their expression.

We, and others, have demonstrated that the long ncRNA CARMN, the host gene for miR-143/145, plays a crucial role as a regulator of vascular smooth muscle cell (SMC) differentiation, vascular integrity, cardiac function, and tissue homeostasis.^1–4^ However, in a variety of cardiovascular diseases, including atherosclerosis and aortic aneurysm, its expression and that of the microRNAs are downregulated in SMCs, leading to pathological vascular remodeling.^1,3^ Given that CARMN transcripts (> 50 transcripts GENECODE V39) and the 2 microRNAs serve distinct and collective functions, we hypothesized that transcriptional activation of the entire set of transcripts is optimal to restore physiological SMC function, health, and integrity. Supporting this, preclinical studies show that nanoparticle delivery of miR-145 can partially reduce atherosclerosis in vivo, highlighting its therapeutic potential.^5^ However, traditional methods such as overexpression require targeting each transcript separately, making them complex and impractical for therapy.

Hence, we sought to use a simpler CRISPR-mediated approach to effectively induce transcription of the CARMN locus (20 kb), leveraging the CRISPR activation system, dCas9-VPR. To identify active transcriptional start sites (TSSs) within the human CARMN locus, we analyzed CAGE-seq (Cap Analysis Gene Expression sequencing; FANTOM5 database) data, H3K4me3 marks in SMCs (ENCODE database) and published GRO-seq (Global Run-on sequencing) data (GSE92375), and found transcriptional activity at 2 regions: (1) upstream of CARMN, named in this study as promoter 1 (P1) and TSS1, and (2) upstream of miR-143/145, referred to as promoter 2 and TSS2 (Figure [Ai]). This finding aligns with mouse studies showing miR-143 and miR-145 are cotranscribed as a bicistronic unit with shared regulatory elements.^4^ For our targeted strategy, we have used the ChopChop and the Broad Institute Web tools to select single-guide RNA (sgRNA) sequences with high on-target rank and minimal off-target activity (Figure [Aii]). Selected sgRNAs were also tested for off-targets using Cas-OFFinder. We first used HEK293T cells to assess transcriptional activation due to their high transfection efficiency and moderate CARMN/miR-143/145 expression, similar to embryonic or endothelial cells.^2,4^ After 48 hours of cotransfecting dCas9-VPR (1.5 μg) with an sgRNA-expressing plasmid (200 ng) targeting a region located at -139 bp upstream of CARMN TSS1 (chr5:149406721), we observed activation of the long ncRNA along with miR-143 and miR-145 (Figure [Bi]). A scrambled sgRNA was used as an experimental control. While targeting promoter 1 enhanced the activation of all locus components, activation of promoter 1 (−216 bp from TSS2, chr5:149428812) induced the expression of miR-143 and miR-145 only, and not CARMN (Figure [Bi]). In this setting, we also observed upregulation of pri-microRNA precursor transcripts (Figure [Bii]). In our previous work, we have investigated whether microRNA precursors could be identified within CARMN isoforms.^3^ RACE and long-read sequencing revealed transcripts ending near the miR-143/145 stem loops. While some shared CARMN’s 5′ end, others had distinct 5′ ends, suggesting the use of alternative promoters. These results support potential coregulation of CARMN and the microRNAs via a shared primary transcript or cis-regulatory enhancer activity.^2^ To evaluate P1-driven transcriptional activation, we measured CARMN expression across exons and junctions (Figure [Ci]). Activation spanned the full transcript, with variations likely due to processing or transcript stability (Figure [Cii]). Having demonstrated proof-of-concept in HEK293T, we next tested transcriptional activation in primary coronary SMCs, as more relevant for modeling human vascular diseases where the CARMN locus plays a critical role. In previous work, we and others have identified CARMN/miR-143/145 role as a regulator of cholesterol-mediated SMC dedifferentiation.^3^

**Figure.**
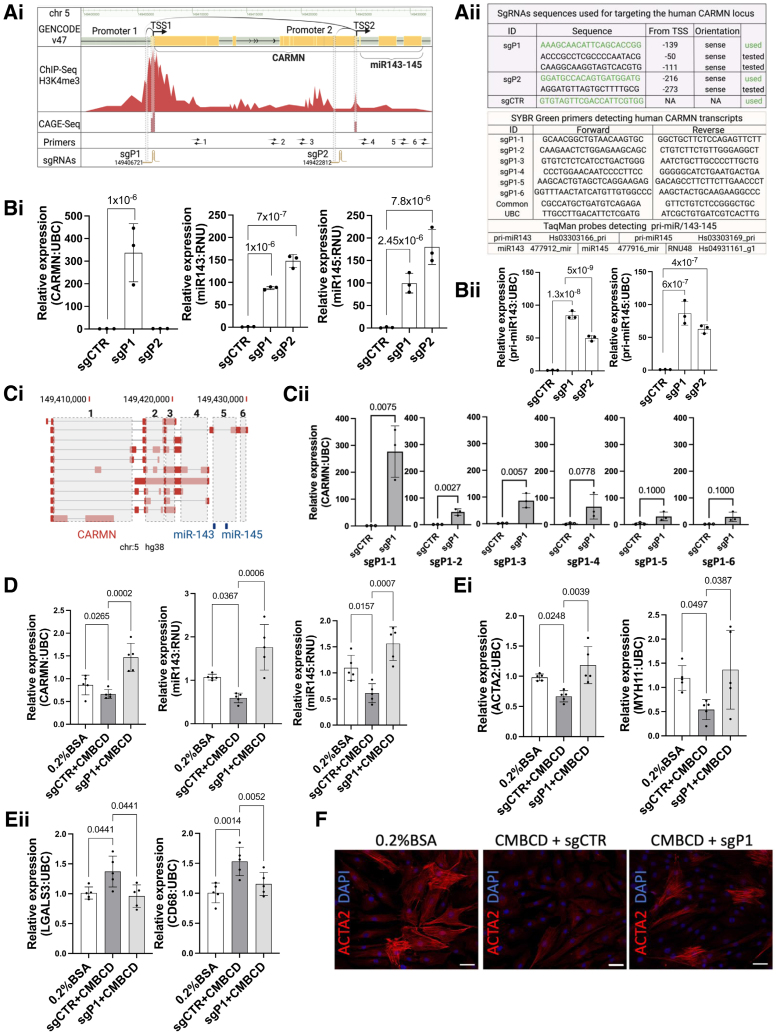
**Effective activation of the human smooth muscle cell (SMC)–specific CARMN/miR-143/145 locus. A**, (**i**) Schematic representation of CARMN/miR-143/145 locus, ChIP-Seq (H3K4me3) tracks, and CAGE-seq (Cap Analysis Gene Expression sequencing) peaks. The figure provides details of the location of the sgRNAs used to activate transcriptional start site (TSS) 1 and TSS2 and the SYBR green primers used in this study; (**ii**) Table displaying the sequences of sgRNA, SYBR green primers, and TaqMan probes used to amplify CARMN and miR-143/145 transcripts. **B**, (**i**) qRT-PCR expression of CARMN/miR-143/145 in HEK293T after transfection with plasmids expressing the VPR system and the sgRNAs activating promoters 1 and 2 (n=3). One-way ANOVA with Bonferroni test for multiple comparisons was used for statistical evaluation; (**ii**) Expression of pri-microRNAs (miRNAs) after activation of promoters 1 and 2 (n=3). One-way ANOVA with Bonferroni test for multiple comparisons was used for statistical evaluation. **C**, (**i**) Simplified representation of exons amplified with primers designed throughout the locus; (**ii**) qRT-PCR expression of different exon regions detected after activation (n=3). Student *t* test with Mann-Whitney *U* test was used for statistical evaluation. **D**, Expression of CARMN/miR-143/145 after delivery of an adenovirus expressing the VPR system and the sgRNA targeting promoter 1 or control virus after cholesterol loading assay with CMBCD (10 µg/mL, 72 hours) or control treatment (0.2% BSA; n=5). One-way ANOVA with Holm-Sidak test for multiple comparisons was used for statistical evaluation. **E**, (**i**) qRT-PCR of SMC markers ACTA2 (smooth muscle actin alpha 2) and MYH11 (myosin heavy chain 11) and (**ii**) proinflammatory markers CD68 and LGALS3 after treatment with CMBCD or control in sgCTR and sgRNA targeting promoter 1 (sgP1) conditions (n=5). One-way ANOVA with Holm-Sidak test for multiple comparisons was used for statistical evaluation. **F**, Immunostaining showing ACTA2 expression after treatment with CMBCD or control in sgCTR and sgP1 conditions. DAPI (4',6-diamidino-2-phenylindole) was used to stain cell nuclei. Scale bar 50 µm. UBC and RNU48 were used as housekeeping controls for qRT-PCR data. Fold changes were calculated by using the 2^−ΔΔCt^ method. Data are presented as bar charts of the SEM with data points for individual replicates (GraphPad 10). Schematics were created using biorender.com.

To model a pathological state, coronary SMCs were cholesterol-loaded with methyl-β-cyclodextrin (10 µg/mL, 72 hours), known to induce downregulation of CARMN locus. We then assessed the effect of CARMN activation using adenoviral VPR delivery with a synthetic sgRNA targeting promoter 1. At 48 hours posttransduction, we observed upregulation of CARMN/miR-143/145 compared with a control virus (sgCTR), suggesting that effective transcriptional activation can be achieved even under propathological conditions (Figure [D]). Further characterization of SMC contractile markers showed increased ACTA2 (smooth muscle actin alpha 2) and MYH11 (myosin heavy chain 11) expression, alongside a reduction in proinflammatory CD68 (cluster of differentiation 68) and LGALS3 (Galectin 3) markers (Figure [E] and [F]), indicating that CARMN locus activation promotes a shift toward a more contractile and less inflammatory phenotype and may play a role in counteracting pathological remodeling. We observed a differing extent of induction between coronary SMCs and HEK293T cells, likely due to baseline expression differences and distinct delivery methods, plasmid transfection in HEK293T, or adenoviral transduction and sgRNA transfection in coronary SMCs. The development of an all-in-one vector incorporating all necessary components for the cis activation of regulatory regions could improve delivery efficiency and enhance therapeutic feasibility in the future. Given the key role of the CARMN/miR-143/145 axis in vascular homeostasis, precise activation in specific cell types using inducible, cell-specific systems will be important. In vivo testing will also be needed to evaluate safety and efficacy. In conclusion, this study establishes a framework for the coordinated and endogenous regulation of complex loci, encompassing both coding and noncoding regions. By targeting key regulatory genomic elements, this approach addresses the limitations of traditional exogenous overexpression vectors and holds promise for clinical translation.

## Article Information

### Acknowledgments

The authors thank Dr Anna Williams, Dr Donal O’ Carroll, and Dr Azzurra De Pace (Edinburgh University), Dr Mauro Giacca (King’s College, London), Dr Igor Ulitsky (Weizmann Institute of Science) for their helpful collaboration, and Dr Joseph Miano (Augusta University) for the insightful suggestions on the manuscript.

### Sources of Funding

The Wellcome Trust Institutional Translational Partnership Award (WT iTPA PIII-090; F. Vacante), the European Research Council Proof-of-Concept Award ACTIVATE (101061472; A.H. Baker), and the British Heart Foundation program grant RG/20/5/34796 (A.H. Baker).

### Disclosures

No potential competing interest was reported by the author(s).

### Supplemental Material

Major Resources Table
